# Customised in vitro model to detect human metabolism-dependent idiosyncratic drug-induced liver injury

**DOI:** 10.1007/s00204-017-2036-4

**Published:** 2017-07-31

**Authors:** Laia Tolosa, Nuria Jiménez, Gabriela Pérez, José V. Castell, M. José Gómez-Lechón, M. Teresa Donato

**Affiliations:** 1 Unidad de Hepatología Experimental, Torre A, Instituto de Investigación Sanitaria La Fe (IIS La Fe), Av Fernando Abril Martorell 106, 46026 Valencia, Spain; 2 Departamento de Bioquímica y Biología Molecular, Facultad de Medicina, Universidad de Valencia, 46010 Valencia, Spain

**Keywords:** Idiosyncrasy, Drug-induced liver injury, CYP, Cell model, Hepatotoxicity mechanisms

## Abstract

Drug-induced liver injury (DILI) has a considerable impact on human health and is a major challenge in drug safety assessments. DILI is a frequent cause of liver injury and a leading reason for post-approval drug regulatory actions. Considerable variations in the expression levels of both cytochrome P450 (CYP) and conjugating enzymes have been described in humans, which could be responsible for increased susceptibility to DILI in some individuals. We herein explored the feasibility of the combined use of HepG2 cells co-transduced with multiple adenoviruses that encode drug-metabolising enzymes, and a high-content screening assay to evaluate metabolism-dependent drug toxicity and to identify metabolic phenotypes with increased susceptibility to DILI. To this end, HepG2 cells with different expression levels of specific drug-metabolism enzymes (CYP1A2, CYP2B6, CYP2C9, CYP2C19, CYP2D6, CYP2E1, CYP3A4, GSTM1 and UGT2B7) were exposed to nine drugs with reported hepatotoxicity. A panel of pre-lethal mechanistic parameters (mitochondrial superoxide production, mitochondrial membrane potential, ROS production, intracellular calcium concentration, apoptotic nuclei) was used. Significant differences were observed according to the level of expression and/or the combination of several drug-metabolism enzymes in the cells created ad hoc according to the enzymes implicated in drug toxicity. Additionally, the main mechanisms implicated in the toxicity of the compounds were also determined showing also differences between the different types of cells employed. This screening tool allowed to mimic the variability in drug metabolism in the population and showed a highly efficient system for predicting human DILI, identifying the metabolic phenotypes associated with increased DILI risk, and indicating the mechanisms implicated in their toxicity.

**Electronic supplementary material:**

The online version of this article (doi:10.1007/s00204-017-2036-4) contains supplementary material, which is available to authorized users.

## Introduction

Drug-induced liver injury (DILI) has a considerable impact on human health and is a major challenge in drug safety assessment. It is the most frequent cause of acute liver failure and a leading reason for the attrition of drug candidates, restriction of use and the post-market withdrawal of approved drugs (Guengerich 2011; Ostapowicz et al. 2002). Preclinical drug safety evaluation aims to minimise potential risks to humans and financial costs. However, early detection of the hepatotoxic potential of drug candidates is hindered by human hepatotoxicity being poorly correlated with regulatory animal toxicity tests, and also by lack of detailed mechanistic information (Olson et al. 2000; Xu et al. 2004). Toxicity predictions are particularly difficult for idiosyncratic DILI, that is, unexpected adverse reactions which occur in susceptible individuals at therapeutic drug doses that are safe for the general population.

The liver’s particular vulnerability to drug toxicity is probably due to its active role in the metabolism of drugs. Metabolism is the major source of the interindividual differences encountered in the pharmacokinetics of drugs and is an indirect determinant of their clinical efficacy and toxicity. Although drug-metabolising reactions often render stable non-toxic metabolites, some drugs are transformed into reactive metabolites that are capable of inducing toxicity (Guengerich 2011; Park et al. 2011). Bioactivation processes have a considerable impact on DILI and are a major concern in drug development.

Considerable variations in the expression levels of both cytochrome P450 (CYP) and conjugating enzymes have been found in human livers (Gomez-Lechon et al. 2007). Genetic polymorphisms, gender, age, hormonal status and drug intake are the key factors responsible for such interindividual differences. Variability in drug-metabolising enzymes is the rule, rather the exception. Indeed each person or population group shows a characteristic metabolic capability and, consequently, a potentially unique susceptibility to DILI. It is reasonable to assume that the individuals with a metabolic phenotype which results in the increased generation of reactive metabolites from a given drug (e.g. high levels of the enzymes involved in drug bioactivation and/or diminished activity of detoxifying enzymes) will pose an increased risk of hepatotoxicity induced by this drug. Conversely, those patients with an accelerated inactivating metabolism will be less sensitive to the drug. In this context, the availability of in vitro strategies capable of reproducing characteristic drug-metabolising phenotypes will be most useful to improve DILI predictions in early drug development stages.

In recent years, different human liver-derived in vitro cell models have been used in drug safety testing (Gomez-Lechon et al. 2014). Among them, liver cell lines, manipulated to express drug-metabolising enzymes, have been proposed to identify the enzymes involved in the metabolism of drugs and for hepatotoxicity predictions (Dambach et al. 2005; Donato et al. 2010, 2013; Vignati et al. 2005; Xuan et al. 2016). We recently reported the utility of HepG2 cells transduced with recombinant adenoviruses to simultaneously express up to five relevant CYPs at levels comparable to those of human hepatocytes in order to test metabolism-dependent hepatotoxicity (Tolosa et al. 2012a, 2013). In another study, remarkable differences were observed in the toxicity of aflatoxin B1, a bioactivable compound, to two sets of HepG2 cells, generated with different combinations of adenoviruses that encode CYPs (Donato et al. 2013). These findings suggest the potential utility of this adenoviral strategy to evaluate the susceptibility of different population groups to drugs metabolised by enzymes with high variability in the human liver (e.g. polymorphic or inducible enzymes).

In the present study, we propose using tailored HepG2 cells, which reproduce specific drug-metabolising enzyme profiles, for safety risk assessments. To this end, cells generated with different combinations of adenoviruses for human CYPs and/or conjugating enzymes (CYP1A2, CYP2B6, CYP2D6, CYP2E1, CYP2C9, CYP2C19, CYP3A4, UGT2B7 and GSTM1) were evaluated for their sensitivity to nine drugs with well-documented metabolism-dependent hepatotoxicity. Drug-induced toxicity was assessed by a high-content screening (HCS) approach that allows the measurement of multiple parameters indicative of cell injury. Our results showed the potential of this cell-based strategy to investigate the mechanisms involved in drug-induced hepatotoxicity, to examine the role of bioactivating and protective/inactivating enzymes in drug toxicity, and to identify metabolic phenotypes that may be associated to increased DILI risk.

## Materials and methods

### Material

Culture media and complements were purchased from GIBCO (Gibco BRL, Paisley, UK). The tested compounds and substrates used for enzyme activity measurements were acquired from Sigma Aldrich (Madrid, Spain). Fluorescent probes tetramethyl rhodamine methyl ester (TMRM), Fluo-4 acetoxymethyl ester (Fluo-4 AM), BODIPY493/503, CellROX Deep Red, MitoSOX red and YO-PRO-1 were obtained from Molecular Probes, Invitrogen (Madrid, Spain). Propidium iodide (PI) and Hoechst 33342 came from Sigma Aldrich.

### Construction of recombinant adenoviruses

Recombinant adenoviruses for several CYP enzymes were developed in our laboratory, as described elsewhere: CYP1A2, CYP2C9 and CYP3A4 (Donato et al. 2010), CYP2C19 and CYP2D6 (Tolosa et al. 2013) and CYP2E1 (Lahoz et al. 2013). The coding sequences of UGT2B7 and GSTM1 were obtained from human liver mRNA through high-fidelity RT-PCR. CYP2B6 cDNA was obtained from cells previously generated in our laboratory (Bort et al. 1999) using the forward and reverse sequences listed in Supplementary Table S1. Purified cDNAs were double-digested with the appropriate restriction enzymes and ligated into the adenoviral pAC/CMVpLpA plasmid (Gomez-Foix et al. 1992), which was previously digested with the same restriction enzymes to provide directional cohesive cloning. The recombinant plasmid pAC/CMVpLpA that contained CYP2E1, UGT2B7 or GSTM1 cDNA (transfer vector) was co-transfected with pJM17 into 293 cells by calcium phosphate/DNA co-precipitation to obtain a recombined adenovirus that expressed the target enzyme (Becker et al. 1994). The resulting viruses were plaque-purified, expanded into a high-concentration stock and titrated by plaque assay, as previously described in detail (Donato et al. 2010).

### Culture of HepG2 cells and adenovirus infection

HepG2 cells (ECACC No. 85011430) were cultured in Ham´s F-12/Leibovitz L-15 (1:1 v/v) supplemented with 7% foetal calf serum, 50 U of penicillin/ml and 50 μg of streptomycin/ml. For subculturing purposes, cells were detached by treatment with 0.25% trypsin/0.02% EDTA at 37 °C. For the adenovirus infection studies, cells were seeded in 96-well plates (5000 cells/well) and adenovirus-containing medium was added 48 h later. After 24 h, cells were transferred to the adenovirus-free medium and cultured for an additional 24-h period prior to the incubations performed with the test chemicals. To optimise the expression of the functional enzymes in the adenovirus-transduced HepG2 (Ad-HepG2) cells, different doses (MOI, multiplicity of infection, defined as plaque formation units per cell) of each adenovirus were tested. Only the subcytotoxic MOI of adenoviruses were used, as confirmed by the previously described MTT test (Tolosa et al. 2012a).

### Incubation of cells with selected compounds

The drugs included in the study were chosen based on previous information about their hepatotoxicity. Information on the potential mechanisms implicated in the toxicity of each compound and the major enzymes involved in their metabolism is summarised in Table 1. HepG2 or Ad-HepG2 (48 h post-infection) cells were exposed for 24 h to eight concentrations of the tested compounds. Each experimental condition was repeated independently three times (with three wells measured each time). The stock solutions of compounds were prepared in DMSO and were conveniently diluted in the culture medium to obtain the desired final concentrations. The final DMSO concentration in the culture medium was 0.5% (v/v), and the control cultures were treated with the same amount of solvent.

**Table 1:** Pharmacological and toxicological information of the drugs used in the study

Drug	Therapeutic class	DILI category^a^	Label^b^	Toxicity^c^	Bioactivation	Enzyme^d^	*C*_max_^e^ (µM)	Concentrations (μM)
Tienilic acid	Diuretic		WD	CB, OS	Yes	CYP2C9*	82.7	25–1200
Troglitazone	Antidiabetic	Severe	WD	MI, OS	Yes	CYP3A4*	6.4	50–400
Perhexiline	Antianginal	Severe	WD	MI, ST	No	CYP2D6	2.2	10–27.5
Diclofenac	NSAID	High concern	WP	MI, AP	Yes	CYP2C9*, CYP3A4*, UGT2B7*	4.2	5–1500
Valproic acid	Antiepileptic	Severe	BW	MI, OS, ST	Yes	CYP2B6*, CYP2C9* (CYP2E1*, CYP2A6*)	166.1	500–12,000
Isoniazid	Antituberculosis	Severe	BW	MI, OS, AP	Yes	CYP2E1*, GSTM1, NAT2	76.6	5000–50,000
Flutamide	Antiandrogen	Severe	BW	MI, OS	Yes	CYP1A2*, CYP2C19*, CYP3A4*	6.2	50–1000
Acetaminophen	Analgesic/antipyretic	DILI reports		OS, AP	Yes	CYP2E1* (CYP1A2*, CYP3A4*) GST	138.9	500–15,000
Amoxicillin/clavulanate	Antibiotic	High concern	AR		No		41/11	8000/4000–12.000/6000

^a^ Gustafsson et al. (2014)

^*b*^ *BW* black box warning, *WD* withdrawn, *WP* warnings and precautions, *AR* adverse reactions (Chen et al. 2011; Walgren et al. 2005)

^c^ Mechanisms of toxicity: *AP* apoptosis, *CB* covalent binding, *MI* mitochondrial impairment, *OS* oxidative stress, *ST* steatosis (Ashrafian et al. 2007; Donato et al. 2009; Fromenty and Pessayre 1997; Rolo et al. 2000; Tolosa et al. 2012b)

^d^ Major enzyme (s) involved in the bioactivation (denoted as *) or detoxification of the drug

^e^ Atienzar et al. (2014), Garside et al. (2014), Tolosa et al. (2012b)

### HCS assay: incubation of fluorescent probes, imaging and analysis

Following treatments, cells were simultaneously loaded with several fluorescent dyes to measure multiple biomarkers of cell toxicity. Different combinations of fluorescent probes were used to identify specific mechanisms of toxicity according to previously described HCS assays (Donato et al. 2012; Tolosa et al. 2015, 2012b). Information about the probes is summarised in Supplementary Table S2.

After incubating with dyes, cells were imaged by the Scan^R system (Olympus, Germany). Dyes were excited, and their fluorescence was monitored at the excitation and emission wavelengths at appropriate filter settings. The collected images were analysed using the Scan^R analysis module, which allows the simultaneous quantification of subcellular structures that are stained by different fluorescent probes. The measured fluorescence intensity was associated with the predefined nuclear and cytoplasmic compartments (Tolosa et al. 2012b).

### HCS data analysis

The concentrations that brought about the 50% reductions in cell viability (IC_50_) in relation to the solvent-treated cells were mathematically calculated from the concentration–effect curves.

The minimal effective concentration (MEC) was defined as the lowest concentration to produce a significant change (*p* ≤ 0.05) in all the analysed parameters compared to the control (solvent-treated) cells. For all the compounds and studied parameters, the MEC led to at least a 20% variation in fluorescence intensity or in the corresponding morphological parameter compared to the untreated cultures. Thus, the toxicity risk (TR) for each compound was also defined as the 100 × *C*_max/_MEC ratio. A 100-fold *C*_max_ scaling factor was considered a physiologically relevant dosing limit for drugs, and a reasonable threshold to differentiate toxic from safe drugs, as previously described (Xu et al. 2008). The lowest MEC found for each compound in any parameter was considered to calculate the TR.

### Data analysis

All the data are expressed as mean ± SEM values and represent triplicate measurements of independent experiments. For statistical analysis real values of control and test compounds were compared. A Student’s *t* test was used for the statistical evaluations calculated with GraphPad Prism vs. 6.1. The chosen significance level was *p* < 0.05.

## Results

### Metabolism-dependent drug toxicity in the cells that overexpress a single CYP

The HepG2 cells infected with different subcytotoxic doses (MOI) of a single AdCYP were generated and resulted in controlled dose-dependent levels of CYP activities (Supplementary Figure S1). For each CYP, high activity values (up to ten-fold over those of the primary human hepatocytes) were reached in the AdCYP-HepG2 cells. The utility of these cells to evaluate metabolism-dependent hepatotoxicity was examined with a set of eight model drugs known to induce marked DILI, and with available information on the major CYP implicated in their bioactivation or detoxication (Table 1). The effects of a range of concentrations of each model compound on cell viability were comparatively examined in both the parental HepG2 cells and the AdCYP-HepG2 cells, and variable levels of the CYP of interest were expressed. The IC_50_ for each drug and cell system are summarised in Table 2. Dose-dependent effects of test compounds on cell viability assessed by PI are shown in Supplementary Figure S2.

**Table 2:** Drug toxicity in HepG2cells transfected with single ADV-CYPs

Drug	ADV	*C*	ADV dose			*C*/x10 ratio
			x1	x4	x10	
Acetaminophen	CYP2E1	>15,000	>15,000	>15,000	10,600	>1.4
Diclofenac	CYP2C9	167	42.1	51.6	15.5	11
	CYP2C19	167	140	158	11.5	15
	CYP3A4	167	126	99	2.8	60
Flutamide	CYP1A2	398	384	261	248	1.4
	CYP2C19	398	344	309	255	1.6
	CYP3A4	398	219	176	145	2.7
Isoniazid	CYP2E1	61,500	52,900	44,500	46,600	1.3
Perhexiline	CYP2D6	22.5	24.4	25.5	27.4	0.8
Tienilic acid	CYP2C9	>1200	>1200	1160	846	>1.4
Troglitazone	CYP3A4	213	86	76	<50	>4.3
Valproic acid	CYP2B6	14,850	13,300	8600	8700	1.7
	CYP2C99	14,850	14,350	15,980	10,070	1.5

The IC50 values for each compound and condition are represented

Troglitazone was more toxic to the cells transduced with AdCYP3A4 than to the control HepG2 cells, and cytotoxicity increased according to CYP3A4 activity, in accordance with the role of CYP3A4 in troglitazone activation (Supplementary Figure 2). Similarly, the toxicity of other bioactivable drugs to HepG2 cells was enhanced upon cell transduction with a single AdCYP: tienilic acid (CYP2C9), isoniazid (CYP2E1), acetaminophen (CYP2E1), valproic acid (CYP2B6 or CYP2C9), flutamide (CYP1A2, CYP2C19 or CYP3A4) and diclofenac (CYP2C9, CYP2C19 or CYP3A4) (Table 2, Supplementary Figure 2). In all cases, an adenovirus dose-dependent increase in drug toxicity was observed, and lower IC_50_ values were obtained in the cells prepared with the largest amount of adenoviruses. Regarding perhexiline, a non-bioactivable hepatotoxic drug, the cells that overexpressed CYP2D6 (the major CYP enzyme involved in its metabolism) showed less sensitivity to perhexiline toxicity than the control HepG2 cells (Table 2).

### Applying the HCS hepatotoxicity assays to the cells that express a single CYP

To increase assay sensitivity and to obtain mechanistic information on drug toxicity, the adenoviral upgraded HepG2 cells were used in combination with the HCS assays, which simultaneously measured the multiple cell endpoints indicative of different mechanisms of toxicity. This mechanism-based strategy was applied to examine the CYP-dependent toxicity of troglitazone, tienilic acid and perhexiline (drugs metabolised mainly by a single CYP, and which have been withdrawn from the market because of their severe hepatotoxicity), and flutamide (metabolised by several CYPs and with a black box warning because of hepatotoxicity). Apoptotic cell death, mitochondrial membrane potential (MMP), ROS and mitochondrial superoxide production, intracellular calcium concentration and, for perhexiline and valproate, lipid overaccumulation, were evaluated. The resulting MEC values are summarised in Supplementary Table S3.

**Fig. 1:**
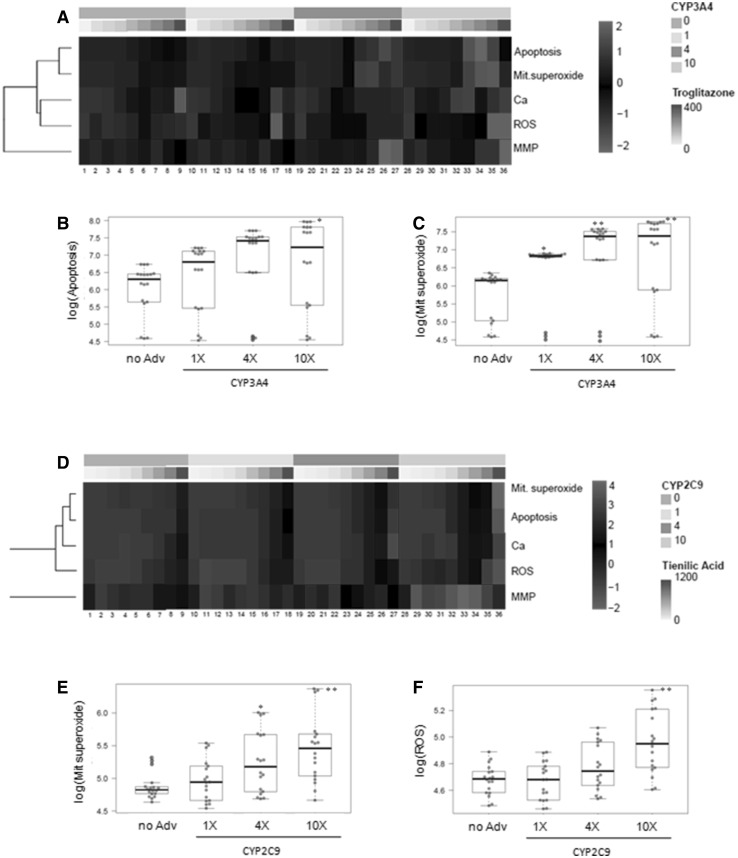
Multiparametric assessment of troglitazone and tienilic acid toxicity in HepG2 cells that express variable levels of a single CYP. Individually, AdCYP-transduced cells were treated for 24 h with increasing concentrations of troglitazone (**a**–**c**) or tienilic acid (**d**–**f**). Then the effects on the apoptotic cell number (apoptosis), mitochondrial superoxide production (Mit. superoxide), intracellular calcium level (Ca), ROS generation (ROS) and mitochondrial membrane potential (MMP) were analysed by HCS. **a** Heatmap of troglitazone (0, 50, 75, 100, 150, 200, 250, 300 and 400 µM) and CYP3A4. Differential effects of troglitazone on apoptosis (**b**) and mitochondrial superoxide production (**c**) according to the level of CYP3A4. **d** Heatmap of tienilic acid (0, 25, 100, 200, 400, 600, 800 and 1200 µM) and CYP2C9. Effects on mitochondrial superoxide (**e**) and ROS production (**f**) depending on the CYP2C9 level. CYP: 0 (non-transduced cells); 1, 4 and 10 correspond to the cells transduced with a single AdCYP to reach the same level, four-fold or ten-fold, respectively, of CYP activity in human hepatocytes. *p < 0.01; **p < 0.001 compared to non-transduced cells (Student’s *t* test)

In addition to reduced cell viability, exposure of HepG2 cells to troglitazone resulted in significant increases in the number of apoptotic cells, ROS generation and calcium levels, whereas weaker effects were observed in MMP and mitochondrial superoxide production (Fig. 1a). All these mechanisms have been described to be key in the hepatotoxicity of troglitazone. As expected, a AdCYP3A4-dose-dependent effect was detected for all the studied parameters, and the maximum effect was obtained in the cells transduced with the highest AdCYP3A4 dose, which was statistically significant when analysing the effects on apoptosis and mitochondrial superoxide production, two specific mechanisms implicated in the toxicity of troglitazone (Fig. 1b, c).

The assessment of the toxicity of tienilic acid to HepG2 cells revealed alterations in all the evaluated parameters, unless MMP, and a marked CYP2C9-dependent toxicity, was observed (Fig. 1d). By way of example, apoptosis and mitochondrial superoxide levels significantly increased in the cells transduced with the highest AdCYP2C9 dose in comparison with the non-transduced cells. This finding suggests increased susceptibility to the toxicity of tienilic acid (Fig. 1e, f).

**Fig. 2:**
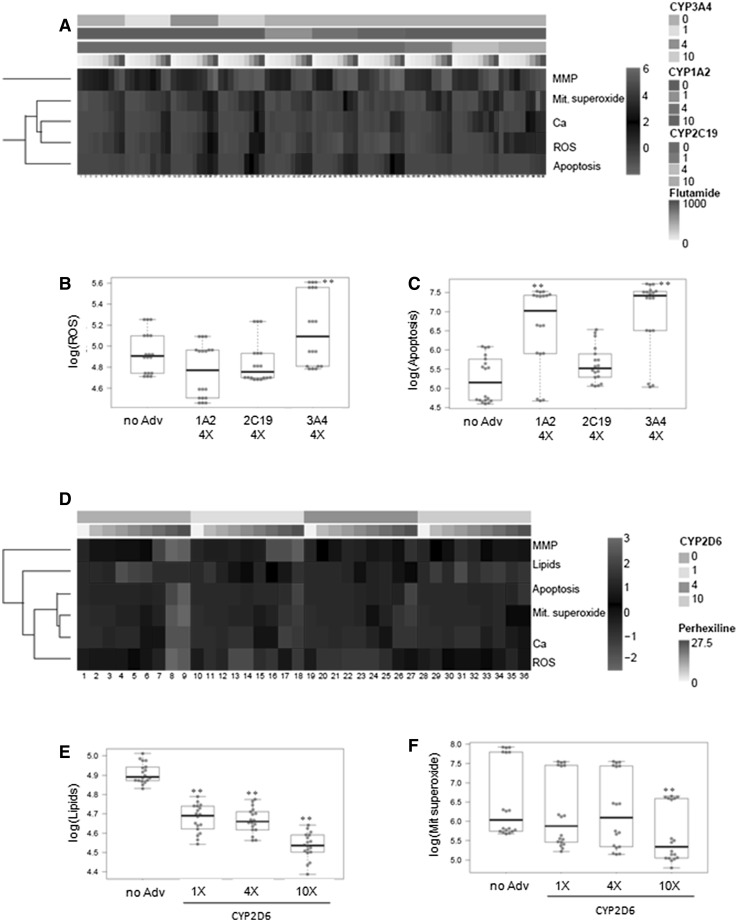
Multiparametric assessment of flutamide and perhexiline toxicity to the HeoG2 cells that express variable levels of a single CYP. Individually, AdCYP-transduced cells were treated for 24 h with increasing concentrations of flutamide (**a**–**c**) or perhexiline (**d**–**f**). Dose-dependent effects on the mitochondrial membrane potential (MMP), ROS generation (ROS), number of apoptotic cells (apoptosis), mitochondrial superoxide production (Mit. superoxide) and intracellular calcium level (Ca) were determined by the HCS analysis. **a** Heatmap of flutamide (0, 50, 75, 100, 125, 250, 500, 750 and 1000 µM) and CYP3A4, CYP1A2 or CYP2C19. Differential effects of flutamide on ROS production (**b**) and induction of apoptosis (**c**) according to the level of CYPs. **d** Heatmap of perhexiline (0, 10, 12.5, 15, 17.5, 20, 22.5, 25 and 27.5 µM) and CYP2D6; neutral lipids accumulation (lipids) was also analysed. Effects on lipid overaccumulation (**e**) and mitochondrial superoxide production (**f**) depending on the CYP2D6 level. CYP: 0 (non-transduced cells); 1, 4 and 10 correspond to the cells transduced with a single AdCYP to reach the same level, four-fold or ten-fold, respectively, of CYP activity in human hepatocytes. **p < 0.001 compared to non-transduced cells (Student’s *t* test)

Different CYPs have been implicated in the hepatotoxicity of flutamide. In the non-transduced cells, significant changes were found for the distinct parameters analysed after treatment with similar concentrations of flutamide. In the AdCYP-HepG2 cells, changes in ROS and apoptotic cell death seemed the most sensitive parameters, although some disparity was observed (Fig. 2a); e.g. the cells transduced with AdCYP3A4 were very sensitive to both ROS production and apoptotic cell death, whereas the significant effect for the cells with CYP1A2 was observed for apoptotic cell death (Fig. 2b, c). In all cases, transduction with AdCYPs resulted in increased toxicity, evidenced by lower MEC values, and the cells that overexpressed CYP1A2 or CYP2C19 were particularly susceptible to flutamide (up to ten-fold lower MEC values compared to the control HepG2 cells) (Supplementary Table S3).

The lowest MEC for perhexiline was observed in the control (non-transduced) HepG2 cells for changes in the intracellular calcium concentration, mitochondrial superoxide production and lipid overaccumulation (Fig. 2d, Supplementary Table S3). The most sensitive parameter for all the conditions was the intracellular calcium concentration. Lipid overaccumulation, a described mechanism of perhexiline-induced toxicity, was detected only in the control cells, even at the lowest concentration used, but not in the cells with increasing CYP2D6 concentrations (Fig. 2e). When studying mitochondrial superoxide production, significant differences were found between the control cells and the cells transduced with different CYP2D6 concentrations (Fig. 2f). The different studied parameters showed that the cells transduced with AdCYP2D6 were less vulnerable to perhexiline-induced hepatotoxicity, which could indicate the participation of this CYP in its metabolism, as previously described in the literature.

### Mechanistic study of metabolism-dependent hepatotoxicity in the cells that express multiple drug-metabolising enzymes

Adenoviral-mediated transduction allows the controllable co-expression of multiple transgenes. Thus, the cells that show specific expression patterns of drug-metabolising enzymes can be easily generated with appropriate mixtures of adenoviruses (Supplementary Figure S3). Different sets of HepG2 cells that emulate metabolic variability in the human liver were prepared to analyse the toxic effects of four bioactivable drugs with hepatotoxicity warnings (valproic acid, acetaminophen, isoniazid and diclofenac) according to the relative activity levels of the major enzymes (CYPs and conjugating enzymes) involved in their metabolism. Supplementary Table S4 summarises the MEC values and the TRs calculated for each compound in the different Adv-cells used.

**Fig. 3:**
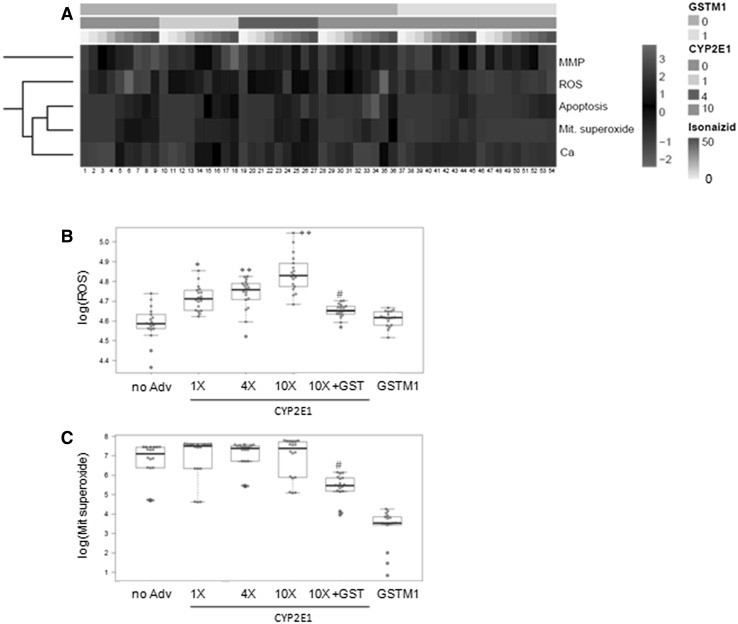
Isoniazid-induced toxicity to the HepG2 cells co-transduced with AdCYP2E1 and AdGSTM1. The HepG2 cells transduced with different amounts of AdCYP2E1, either alone or in combination with AdGSTM1, were treated for 24 h with isoniazid (0, 5, 10, 20, 30, 35, 40, 45 and 50 mM). **a** Heatmap showing isoniazid toxicity. Dose-dependent effects on the mitochondrial membrane potential (MMP), ROS generation (ROS), number of apoptotic cells (apoptosis), mitochondrial superoxide production (Mit. superoxide) and intracellular calcium level (Ca) were determined by the HCS analysis. Differential effects on ROS (B) and mitochondrial superoxide production (C), depending on the level and combination of enzymes, are shown. CYP2E1: 0 (non-transduced cells); 1, 4 and 10 (cells transduced to reach the same level, four-fold or ten-fold, of CYP2E1 activity in human hepatocytes, respectively); GSTM1: 0 (non-transduced cells) and 1 (cells transduced to reach the same GST activity as human hepatocytes). *p < 0.01, **p < 0.001 compared to non-transduced cells; #*p* < 0.01 compared with and without GSTM1 (Student’s *t* test)

An evaluation of the different cell endpoints revealed that isoniazid was more toxic to the cells transduced with AdCYP2E1 than to normal HepG2 (Fig. 3a). Toxicity increased according to the AdCYP2E1 load, which evidences the role of this enzyme in the metabolic activation of isoniazid. The observed CYP2E1-mediated potentiation of the isoniazid toxicity in HepG2 cells was abolished by the simultaneous transduction with AdGSTM1 (Fig. 3A). In fact comparable effects were induced by isoniazid in the AdCYP2E1 and AdGSTM1 co-transduced cells and in the control HepG2 cells (that lack CYP2E1 activity), and even for the most sensitive parameters to CYP2E1 overexpression (e.g. number of apoptotic nuclei, mitochondrial superoxide levels, intracellular calcium concentration, ROS generation). When specifically comparing the effects on ROS and mitochondrial superoxide production, significant differences were found not only between the control cells and the cells transduced with AdCYP2E1, but also between the AdCYP2E1 cells and those with AdCYP2E1 and AdGSTM1 (Fig. 3b, c).

**Fig. 4:**
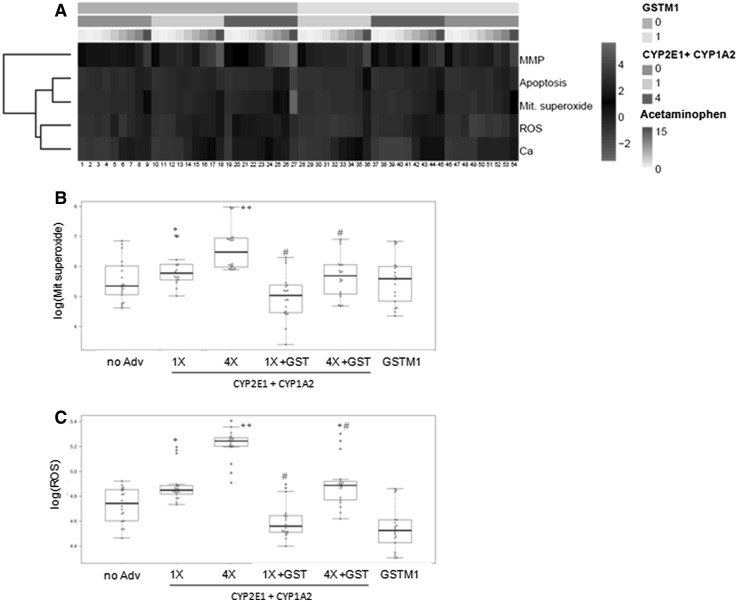
Acetaminophen-induced toxicity to the HepG2 cells co-transduced with AdCYP2E1, CYP1A2 and AdGSTM1. The HepG2 cells transduced with a mixture of AdCYP2E1 and AdCYP1A2, in combination or not with AdGSTM1, were treated for 24 h with acetaminophen (0, 0.5, 1, 2, 4, 6, 8, 10 and 15 mM). **a** Heatmap of acetaminophen toxicity in the cells transduced with different adenoviruses. Dose-dependent effects on the mitochondrial membrane potential (MMP), ROS generation (ROS), number of apoptotic cells (apoptosis), mitochondrial superoxide production (Mit. superoxide) and intracellular calcium level (Ca) were determined by the HCS analysis. Differential effects on mitochondrial superoxide (**b**) and ROS production (**c**) according to the level or combination of enzymes are shown. CYP2E1 + AdCYP1A2: 0 (non-transduced cells); 1, 4 and 10 (cells co-transduced to reach the same level, four-fold or ten-fold, of each CYP activity in human hepatocytes, respectively); GSTM1: 0 (non-transduced cells) and 1 (cells transduced to reach the same GST activity as human hepatocytes). *p < 0.01, **p < 0.001 compared to non-transduced cells; #*p* < 0.001 compared with and without GSTM1 (Student’s *t* test)

Exposure of HepG2 cells to non-lethal concentrations of acetaminophen (<15 mM) produced significant alterations to several parameters, which is indicative of cell damage (nuclear changes, mitochondrial superoxide, intracellular calcium, ROS levels), and these effects were stronger in the cells that overexpressed CYP2E1 (Fig. 4a). The toxicity of acetaminophen was also evaluated in the cells prepared with different combinations of the adenoviral constructs of CYP2E1, CYP1A2 (another CYP involved in acetaminophen bioactivation) and GSTM1 (greatly involved in the detoxification of reactive metabolites). Co-transduction with AdCYP2E1 and AdCYP1A2 resulted in a significantly increased toxicity compared to the cells single-transduced with AdCYP2E1 (Fig. 4b, c). This was particularly evident for the cells treated with intermediate doses of adenoviruses; e.g. 0.5 mM acetaminophen induced significant changes in the ROS generation and calcium levels in co-transduced cells, whereas much higher concentrations (4–6 mM) were needed to significantly alter these parameters in the AdCYP2E1-HepG2 cells. These toxic effects were statistically and significantly reduced by the concomitant overexpression of GSTM1 (AdCYP2E1 + AdCYP1A2 vs. AdCYP2E1 + AdCYP1A2 + AdGSTM1), which agrees with the role of each enzyme in acetaminophen bioactivation/detoxification (Fig. 4b, c).

**Fig. 5:**
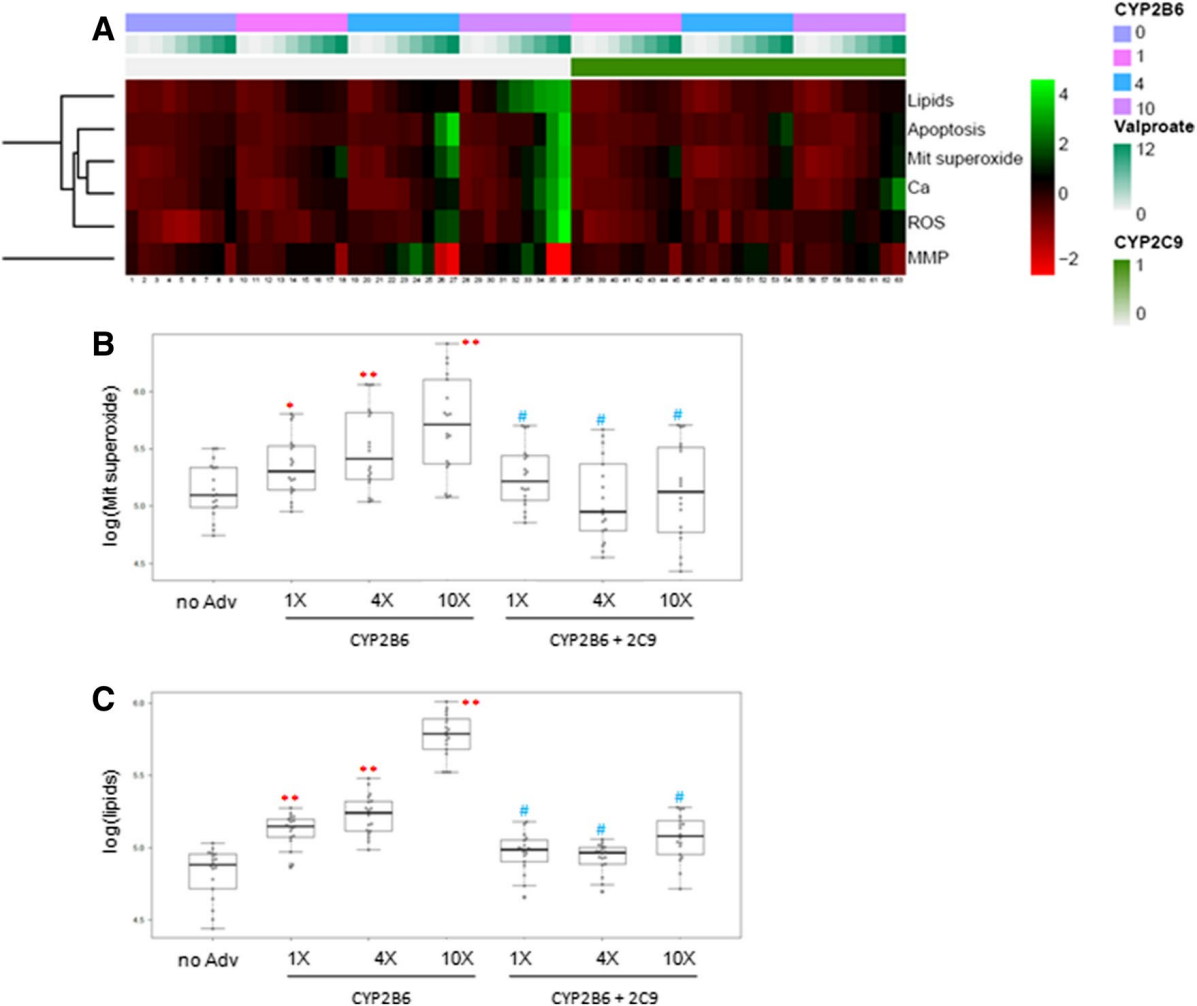
Valproate-induced toxicity to the HepG2 cells co-transduced with AdCYP2B6 and CYP2C9. The HepG2 cells transduced with different amounts of AdCYP2B61, either alone or in combination with AdCYP2C9, were treated for 24 h with valproate (0, 0.5, 1, 2, 4, 6, 8, 10 and 12 mM). **a** Heatmap of valproate toxicity. Dose-dependent effects on the neutral lipid content (lipids), number of apoptotic cells (apoptosis), mitochondrial superoxide production (Mit. superoxide), intracellular calcium level (Ca), ROS generation (ROS), and mitochondrial membrane potential (MMP) were determined by the HCS analysis. Differential effects on mitochondrial superoxide production (**b**) and lipid overaccumulation (**c**) in the cells that expressed different levels of CYP2B6 and CYP2C9 are exemplified. CYP2B6: 0 (non-transduced cells); 1, 4 and 10 (cells transduced to reach the same level, four-fold or ten-fold, of CYP2B6 activity in human hepatocytes, respectively); CYP2C9: 0 (non-transduced cells) and 1 (cells transduced to reach the same CYP2C9 activity as human hepatocytes). *p < 0.01, **p < 0.001 compared to non-transduced cells; #*p* < 0.001 comparing cells expressing different levels of CYP2B6 with and without CYP2C9 (Student’s *t* test)

The evaluation of the toxicity of valproate by means of HCS demonstrated that lipid overaccumulation, apoptotic cell death and mitochondrial superoxide production were the most sensitive parameters to be altered after exposure to it, although calcium homoeostasis and apoptotic cell death were also significantly induced (Fig. 5a). When cells were transduced with increasing concentrations of an adenovirus that encodes for CYP2B6, valproate-induced hepatotoxicity significantly increased (Fig. 5b, c), which was partially reverted in the presence of CYP2C9. This scenario indicates the participation of these enzymes in the bioactivation and detoxification of valproate, respectively.

**Fig. 6:**
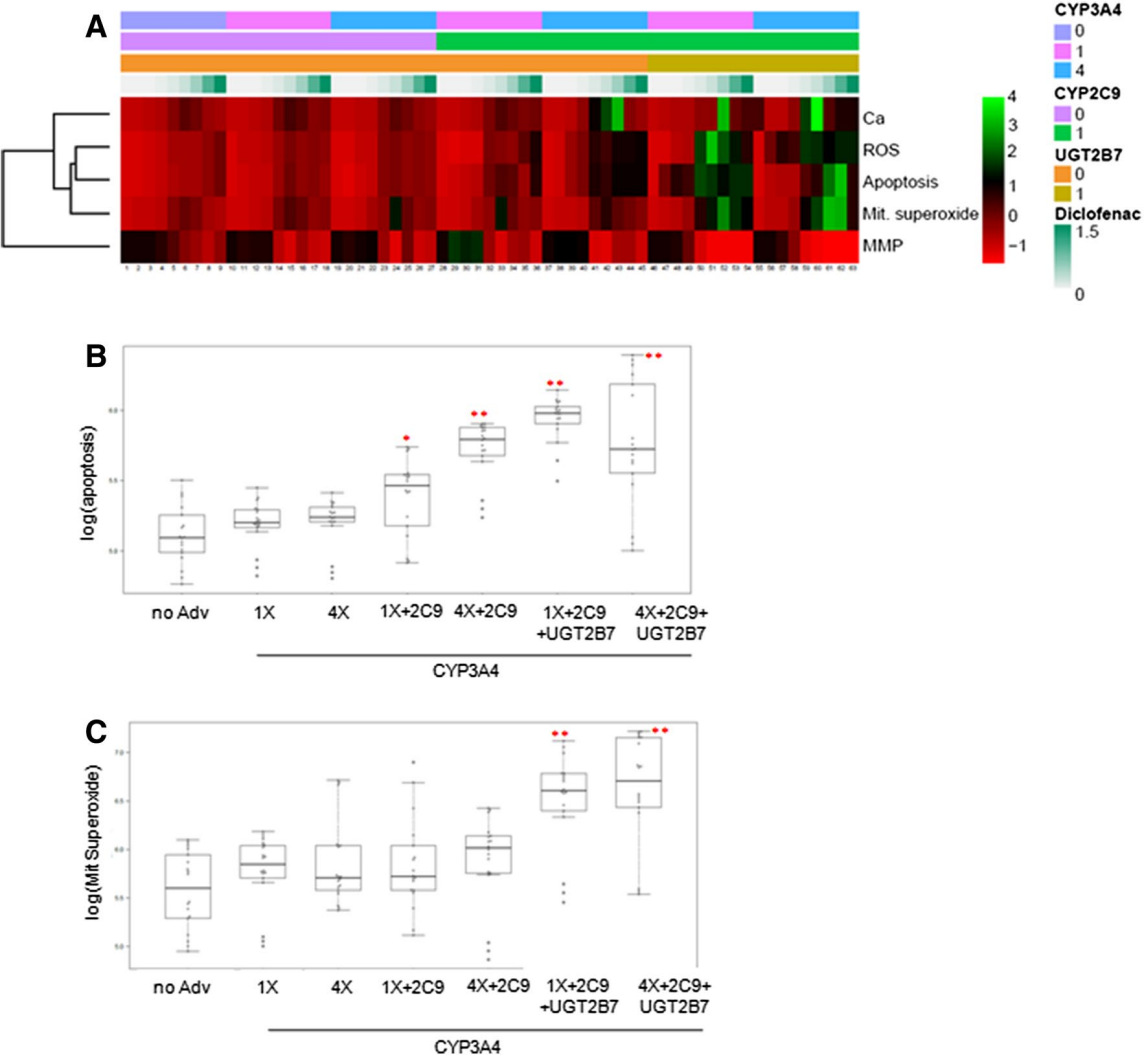
Diclofenac toxicity to the HepG2 cells co-transduced with AdCYP3A4, AdCYP2C9 and AdUGT2B7. The HepG2 cells transduced with different amounts of AdCYP3A4, either alone or in combination with AdCYP2C9 and/or UGT2B7, were treated for 24 h with diclofenac (0, 5, 10, 50, 125, 250, 500, 1000 and 1500 µM). **a** Heatmap of diclofenac-induced hepatotoxicity. Dose-dependent effects on the intracellular calcium level (Ca), ROS generation (ROS), number of apoptotic cells (apoptosis), mitochondrial superoxide production (Mit. superoxide) and mitochondrial membrane potential (MMP) were determined by the HCS analysis. Differential effects on apoptosis (**b**) and mitochondrial superoxide production (**c**) in the cells that expressed different levels, or a combination of enzymes, are shown. CYP3A4: 0 (non-transduced cells); 1, 4 and 10 (cells transduced to reach the same level, four-fold or ten-fold, of CYP3A4 activity in human hepatocytes, respectively); CYP2C9: 0 (non-transduced cells) and 1 (cells transduced to reach the same CYP2C9 activity as human hepatocytes); UGT2B7: 0 (non-transduced cells) and 1 (cells transduced to reach the same UGT2B7 activity as human hepatocytes). *p < 0.01, **p < 0.001 compared to non-transduced cells (Student’s *t* test)

Diclofenac-induced hepatotoxicity was studied in detail in the cells transduced with different combinations of adenoviruses. The most marked changes were observed in the cells transduced with a combination of adenovirus for CYP3A4 and CYP2C9 or UGT2B7, which indicates that the CYP-mediated oxidation and glucuronidation of diclofenac play an important role in its hepatotoxicity (Fig. 6a). Conversely, when cells were transduced with CYP3A4 in the presence of CYP2C19, toxicity diminished compared with the AdCYP3A4-HepG2 cells, which indicates that CYP2C19 could play a role in detoxification (Supplementary Table S4). Although different parameters were altered, mitochondrial superoxide and ROS production seemed to be the most sensitive ones. MMP and apoptotic cell death, these being described mechanisms of diclofenac-induced toxicity, also significantly changed in the cells transduced with distinct combinations of adenoviruses that encode for CYP3A4, CYP2C9 and/or UGT2B7. A significant increase in apoptotic cells and mitochondrial superoxide production was observed in the cells transduced with both CYP3A4 and 2C9. This increase became more evident with the addition of UGT2B7, which suggests the participation of these enzymes in the bioactivation of diclofenac (Fig. 6b, c).

Finally, we explored the toxicity of the amoxycillin/clavulanate combination, one of the most commonly implicated agents in severe idiosyncratic liver injury. Unlike other studied drugs, the hepatotoxicity of amoxycillin/clavulanate has not been related to CYP metabolism. As expected, when amoxycillin/clavulanic acid-induced toxicity was comparatively studied in cells transduced with different combinations of adenoviruses, no significant differences were observed for any analysed parameter. Although the incubation of cells with amoxycillin/clavulanic acid produced a significant increase in ROS production and a rise in the intracellular calcium levels, no differential effects were observed in the Ad-HepG2 and HepG2 cells (Supplementary Figure S4).

### Drug safety predictions

**Fig. 7:**
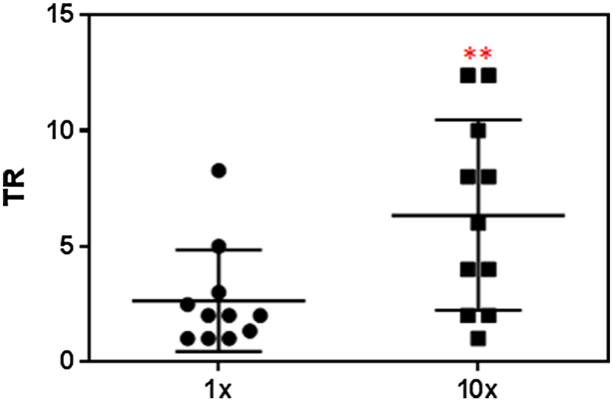
Differences in the TR depending on the AdCYP dose. The TR was calculated as the 100 × *C*_max_/MEC ratio and was normalised by the TR value of the HepG2 cells (**p = 0.002, paired *t* test)

The TR for each drug and cell system (with or without adenoviral transduction) was calculated to understand the significance of the in vitro effects (Supplementary Tables S3 and S4). This index compares the lower drug concentration capable of inducing damage in the in vitro system and the drug concentration reached in a clinical setting. As expected, all the bioactivable drugs obtained a higher TR in the HepG2 cells individually transduced with the CYP involved in their bioactivation than in the non-metabolically competent HepG2 cells, whereas this trend was not observed for the non-bioactivable drugs (perhexiline and amoxycillin/clavulanic). In order to better interpret the possible influence of variability in the phenotypic CYP expression on drug toxicity, Fig. 7 compares the TR obtained in the cells transduced with the low AdCYP dose (the equivalent to CYP activity in human hepatocytes) or with the high dose (to reach a ten-fold higher expression than in hepatocytes). The higher TR values found in the cells with the highest CYP activity level indicate the major participation of the specific CYP in drug bioactivation, which suggests a likely increased toxicity risk in those patients with higher activity levels than those in the general population.

## Discussion

Integrated approaches by means of a panel of engineered cell lines, where each one permanently over expresses an individual CYP enzyme, have been proposed to discriminate hepatotoxic compounds and to identify the role of specific CYPs in the activation and/or the inactivation of drug candidates (Dambach et al. 2005; Greer et al. 2010; Hashizume et al. 2010; Thompson et al. 2012; Xuan et al. 2016). Techniques for stable CYP transfection do not allow the controlled expression of the enzyme, and generated cells often present very high or very low activity levels compared to those in hepatocytes, which seriously impairs the in vivo relevance of the obtained results (Donato et al. 2013; Frederick et al. 2011; Kwon et al. 2014). Moreover, a potential relationship between the enzyme activity level and drug toxicity cannot be examined in these cells. Alternatively, adenovirus-mediated transduction allows cells to be generated with the desired activity levels of the enzyme of interest. We previously used a single dose of adenoviruses to confer drug-metabolizing capability to reproduce the characteristic P450 activity levels pattern of human hepatocytes (Tolosa et al. 2012a, 2013) to study the toxicity of bioactivable compounds. Adenoviral-mediated transduction is a simple and highly reproducible procedure for efficiently delivering multiple genes into cultured cells and for obtaining cells with the desired levels of several enzyme activities. This strategy opens up the possibility of exploring the role of enzymes with high interindividual variability in the human liver (i.e. polymorphic or inducible enzymes) in the bioactivation of drugs, and to comparatively evaluate the susceptibility of the different population groups (i.e. extensive or poor metabolisers) to liver toxicity induced by the drug.

In the present study, we explored the feasibility of the combined use of HepG2 cells transduced with adenoviruses that encode drug-metabolising enzymes and a HCS-based multiparametric testing strategy to evaluate metabolism-dependent drug toxicity and to identify the metabolic phenotypes with increased susceptibility to DILI. Our results evidenced that when HepG2 cells that express a single CYP enzyme were used, the sensitivity of cells to bioactivable drugs was enhanced in parallel to the activity of the enzyme involved in the generation of reactive species (e.g. tienilic acid and CYP2C9, troglitazone and CYP3A4, isoniazid and CYP2E1, valproic acid and CYP2B6, acetaminophen and CYP2E1; flutamide and CYP1A2, CYP2C19 or CYP3A4, or diclofenac and CYP2C9, CYP2C19 or CYP3A4). However, the toxicity of the drugs actively metabolised to non-toxic metabolites was lower in the cells that displayed the highest CYP activity level (e.g. perhexiline and CYP2D6) (Table 2). Although the HepG2 cells manipulated to express high CYP levels have been previously proposed for toxicity assessments (Hashizume et al. 2010; Lahoz et al. 2013; Vignati et al. 2005; Xuan et al. 2016), this is the first attempt to relate the CYP activity level expressed by cells and the toxic effects induced by drugs. The toxicity of tested drugs was estimated as not only reductions in cell viability, but also as alterations in several parameters that are indicative of cell damage via different mechanisms (mitochondrial dysfunction, oxidative stress, impairment of calcium homoeostasis, lipid accumulation or apoptosis) (Figs. 1, 2, 3, 4, 5, 6 and Supplementary Table S3).

The toxicity of tienilic acid and troglitazone, two known examples of market withdrawals due to drug hepatotoxicity (Walgren et al. 2005), to HepG2 cells was dependent on the activity levels of CYP2C9 and CYP3A4, respectively. According to previously reported mechanisms of toxicity (Nishiya et al. 2008; Okuda et al. 2010), both drugs induced marked increases in intracellular calcium, the number of apoptotic nuclei, and ROS and mitochondrial superoxide generation. These effects were more pronounced in the cells that expressed the highest levels of CYP activities. Similarly, the toxic effects found in the cells exposed to flutamide completely coincided with those reported in the literature (Ball et al. 2016; Kashimshetty et al. 2009). This drug, which is widely used to treat prostate cancer, received a black box warning label because of rare episodes of idiosyncratic DILI (Walgren et al. 2005). Although the precise mechanism of flutamide-induced liver damage in susceptible patients remains unknown, mitochondrial dysfunction and oxidative stress are likely to be involved (Kashimshetty et al. 2009). Flutamide hepatotoxicity has been related to bioactivation by CYPs, and the formation of several reactive intermediates during the CYP1A2-, CYP2C19- and CYP3A4-mediated oxidations of flutamide and its metabolites has been reported (Kang et al. 2008). The additional mitochondrial effects of 2-hydroxyflutamide, compared with its parent drug, have been related to idiosyncratic DILI in flutamide-treated patients (Ball et al. 2016). We previously reported that flutamide was more toxic to upgraded Ad-HepG2 cells (co-expressing CYP1A2, CYP2C9, CYP2C19, CYP2D6 and CYP3A4 activity levels equivalent to human hepatocytes) than to parental HepG2 cells (Tolosa et al. 2013). In the present study, we confirmed the role of CYP1A2, CYP2C19 and CYP3A4 in metabolic bioactivation of the drug. Moreover, the differences observed in the effects induced by flutamide, which depend on the overexpressed CYP and its levels (Fig. 2a–c and Supplementary Table S3), could be due to the specific metabolites generated by each CYP, which could produce differential cell toxicity. Perhexiline, an antianginal agent, was removed from the market in many countries worldwide in the 1980s for its hepatotoxicity, but continued to be prescribed in some countries (Ashrafian et al. 2007). Perhexiline hepatotoxicity incidence is low, but can be severe and result in hepatic cirrhosis, and even death. Perhexiline clearance is determined by the hydroxylation catalysed by polymorphic CYP2D6, and it has been recognised that its adverse effects are more likely to occur in CYP2D6 poor metabolisers patients than in extensive metabolisers (Barclay et al. 2003). As expected, perhexiline proved more toxic to HepG2 cells with marginal CYP2D6 activity, and lipid accumulation, intracellular calcium and mitochondrial superoxide were the most sensitive parameters. The patients treated with perhexiline can develop steatohepatitis lesions, and impaired mitochondrial function, lipid accumulation and increased calcium levels have been previously observed in hepatocytes exposed to this drug (Robin et al. 2008). Our proposed strategy generally seems a simple useful screening tool to identify metabolism-dependent toxicity and to provide mechanistic insights into drug-induced hepatotoxicity.

One major limitation of the cells that express a single enzyme is that they show considerable transfected enzyme activity (i.e. a particular CYP), but the expression of other drug-metabolising enzymes (i.e. other CYPs, conjugating enzymes) is generally very low. Thus, the cells present an altered activation/detoxification balance, and two-step drug bioactivation (e.g. sequential oxidations by different CYPs) cannot be properly predicted. The use of stable cell lines that co-express a CYP and a phase II enzyme (e.g. CYP1A1 and GSTP1) have been reported to investigate the bioactivation and detoxification of carcinogens (Kabler et al. 2009). However, constructing cells that permanently express several drug-metabolising enzymes is very difficult and, in practical terms, the co-transfection of more than two enzymes is unfeasible. In contrast, recombinant adenoviruses allow the generation of cells that transiently express controllable levels of multiple enzymes (Tolosa et al. 2013). Hence, by using an appropriate combination of adenoviruses, it is possible to prepare cells with the desired enzyme profile. By adopting this strategy, we obtained cells with variable levels of different combinations of CYPs, UGT2B7 and/or GSTM1 to explore the toxicity of four model drugs, namely isoniazid, acetaminophen, valproate and diclofenac.

Isoniazid is a widely used first-line antituberculosis drug that has been associated with idiosyncratic DILI and has received a black box warning (Walgren et al. 2005). Isoniazid hepatotoxicity incidence is relatively high compared to idiosyncratic reactions induced by other drugs, and different host factors, including polymorphic variants in drug-metabolising enzymes, have been analysed as potential determinants of susceptibility to isoniazid. Among them, high CYP2E1 levels and poor GST activity have been suggested as positive risk factors (Lee et al. 2013; Srivastava et al. 2010). Accordingly, when we tailored cells to express increasing CYP2E1 concentrations, a rise in the intracellular calcium concentration and ROS production was observed, yet isoniazid-induced hepatotoxicity reduced when GSTM1 was added to these cells. Similarly, our results showed an increased toxicity of acetaminophen in the cells with overexpressed variable levels of CYP2E1 and CYP1A2, whereas the effects were reverted in the presence of GSTM1 (Fig. 3). These results are in agreement with the high sensitivity to acetaminophen found in Ad-HepG2 cells expressing high CYP1A2 and CYP2E1 activities (Gomez-Lechon et al. 2017). Acetaminophen is considered a safe drug if taken at therapeutic doses, but an overdose produces liver damage that can be fatal. CYP1A2 and CYP2E1 have been implicated mainly in the conversion of acetaminophen into NAPQI, its highly reactive metabolite, which is detoxified through binding to GSH (Baillie and Rettie 2011). After exposure to valproic acid, we observed alterations in the mitochondrial parameters in those cells that harboured high levels of CYP2B6. These effects were partially reduced in the cells that co-expressed CYP2B6 and CYP2C9 (Fig. 5). Although the mechanism of valproic acid hepatotoxicity is still not well understood, bioactivation into 4-ene-valproic acid, a mitochondrial toxicant, has been proposed to play a role. CYP2B6 and CYP2C9 are involved in 4-ene-valproic acid formation, a minor route of the valproic acid metabolism, but they also mediate valproic acid hydroxylations to form inactive metabolites (Ghodke-Puranik et al. 2013). Then, valproic acid-associated hepatotoxicity could depend on the relative expression of both enzymes. Our results suggest that strong CYP2B6 activity contributes to valproic acid toxicity, whereas CYP2C9 activity might have a protective effect. In fact, idiosyncratic valproic acid toxicity in paediatric patients has been linked to several risk factors, including the concurrent administration of the CYP2B6-inducing drug phenobarbital (Bryant and Dreifuss 1996) or low function CYP2C9 polymorphisms (Nagy et al. 2015). The use of adenoviral-mediated transduced cells revealed a CYP3A4 activity-dependent diclofenac toxicity potentiated by CYP2C9 and, to a great extent, by UGT2B7 (Fig. 5). In a previous study using Ad-HepG2, we showed the role of CYPs in diclofenac toxicity (Tolosa et al. 2013). Here, we evidenced that not only the metabolism of diclofenac by CYP2C9 and CYP3A4, but also glucuronidation by UGT2B7 is critical for its hepatotoxicity due to the formation of reactive metabolites (Tang 2003). The fact that these enzymes showed wide interindividual variability could contribute to patients’ different susceptibility to idiosyncratic hepatotoxicity induced by diclofenac (den Braver et al. 2016).

Current cell-based models for the detection of drug-induced liver injury include primary hepatocytes, hepatocyte-like cells derived from pluripotent stem cells or liver cell lines that express drug-metabolism enzymes. However, in practical terms, the capability of these systems to predict human toxicity is limited for various segments of the population with particular profiles of drug-metabolising enzymes (i.e. genetic polymorphisms). Kwon et al. (2014) developed a cell-based platform that allows control over drug metabolism by manipulating the expression levels of distinct drug-metabolising enzymes. This screening tool has been applied to a few model compounds, although only the effects on cell viability were evaluated. In contrast, our system combines both the control of the expression levels of specific drug-metabolism enzymes by mimicking the variability of expression among the population, and the assessment of a panel of pre-lethal mechanistic parameters, which increases the system’s efficacy in predicting human DILI. On the basis of the high TR values obtained in the cells that overexpressed high CYP levels, the potential associations found between specific CYP phenotypes and the risk of liver damage associated with the therapeutic use of some drugs reported to cause severe hepatotoxicity could have been anticipated (e.g. CYP2C9 and tienilic acid, CYP3A4 and troglitazone).

In summary, our study provides evidence for the potential utility of Adv-HepG2 cells with different levels of expression of relevant drug-metabolising enzymes to study metabolism-dependent hepatotoxicity and to identify the specific enzymes that contribute to the toxicity pattern of a particular drug. In combination with HCS-based multiparametric assessments, this cell model is particularly relevant for mechanistic studies. In line with the growing interest in developing systems that allow both screening and providing information on the mechanisms implicated in hepatotoxicity, our mechanistic approach could cover both aspects. In the near future, our proposal could be a useful tool for hepatotoxicity in vitro predictions in early drug development by providing relevant information to understand the mechanisms that underlie idiosyncratic DILI.

## Electronic supplementary material

Below is the link to the electronic supplementary material.

Supplementary material 1 (DOCX 1733 kb)

Supplementary material 2 (DOCX 1170 kb)

## Abbreviations

CYP: Cytochrome P450
DILI: Drug-induced liver injury
HCS: High-content screening
MEC: Minimum effective concentration
MMP: Mitochondrial membrane potential
MOI: Multiplicity of infection
TR: Toxicity risk
