# Supplementary material for: Customised in vitro model to detect human metabolism-dependent idiosyncratic drug-induced liver injury
Source: Arch Toxicol. 2017 Jul 31;92(1):383–99. doi: 10.1007/s00204-017-2036-4 (PMC5773651; doi:10.1007/s00204-017-2036-4)
Supplement: Supplementary file 1 — Supplementary material 1 (DOCX 1733 kb) [file 204_2017_2036_MOESM1_ESM.docx]

**Supplementary Information**

**Supplementary Materials and Methods**

**Assessment of drug-metabolizing activities in adenovirus transduced cells.**

HepG2 cells were seeded at a density of 2.5 x 10^4^ cells/cm^2^ in 24-well plates. Forty-eight hours later, cells were infected with different sub-cytotoxic amounts (MOI, multiplicity of infection) of adenoviruses for CYPs (Ad-CYP1A2, Ad-CYP2B6, Ad-CYP2C9, Ad-CYP2C19, Ad-CYP2D6, Ad-CYP2E1, Ad-CYP3A4) or fase II enzymes (GSTM1 or UGT2B7), alone or in combination, for 24 h. Then cells were shifted to the adenovirus-free medium and cultured for an additional 24-hour period.

Drug-metabolizing activities were assessed 48 h post-adenoviral infection. CYP activities were measured by incubating cell monolayers with a cocktail of selective substrates for individual enzymes: 10 μM phenacetin (CYP1A2), 10 μM bupropion (CYP2B6), 10 μM diclofenac (CYP2C9), 50 μM mephenytoin (CYP2C19), 10 μM bufuralol (CYP2D6), 50 μM chlorzoxazone (CYP2E1) and 5 μM midazolam (CYP3A4). Formation of the corresponding metabolites was quantified by HPLC tandem mass spectrometry (HPLCMS/MS) as previously described in detail (Gomez-Lechon et al. 2012). UGT2B7 activity was assayed by incubating cells with 600 μM naloxone, following quantification of naloxone 3-glucuronide by HPLC-MS/MS (Donato et al. 2010b Anal Bioanal Chem). GST activity was assayed by incubating the S9 fractions obtained from the cells with 1 mM CDNB as described elsewhere (Donato et al. 2015).

**References supplementary material and methods**

Gomez-Lechon, M. J., Lahoz, A., Castell, J. V., and Donato, M. T. (2012). Evaluation of cytochrome P450 activities in human hepatocytes in vitro. Methods Mol. Biol. 806, 87–97.

Donato, M. T., Montero, S., Castell, J. V., Gomez-Lechon, M. J., and Lahoz, A. (2010). Validated assay for studying activity profiles of human liver UGTs after drug exposure: inhibition and induction studies. Anal. Bioanal. Chem. 396, 2251–2263.

Donato, M. T., Tolosa, L., and Gomez-Lechon, M. J. (2015). Culture and functional characterization of human hepatoma HepG2 cells. Methods Mol. Biol. 1250, 77–93.

**Supplementary Tables**

**Supplementary Table S1. Oligonucleotides used to clone the genes**

| **Gene** | **Ref Seq** | **Oligonucleotide (5' to 3')** |
| --- | --- | --- |
| *CYP2B6* | NM_000767 | *Up-BamHI: CAG GGATCC CAGACCAGGACCATGGAA* |
|  |  | *Dn-BamHI: TTT GGGATCC TTCCCTCAGCCCCTTCAG* |
| *UGT2B7* | NM_001074.2 | *Up-KpnI: CAG GGTACC AGGATGTCTGTGAAATGGACTTCA* |
|  |  | *Dn-HindIII: CAG GGTACC AGGATGTCTGTGAAATGGACTTC* |
| *GSTM1* | NM_000561.3 | *Up-BamHI: CAG GGATCC ACCATGCCCATGATACTGGGGT* |
|  |  | *Dn-XbaI: CAG TCTAGA CTACTTGTTGCCCCAGACAGCC* |

**Supplementary Table S2. Fluorescent probes used for the hepatotoxicity HCS assay**

| Fluorescent probe | Manufacturer | Reference | Final concentration  (µM) | λ_excitation_  (nm) | λ_emission_  (nm) | Parameter |
| --- | --- | --- | --- | --- | --- | --- |
| BODIPY493/503 | Molecular Probes | D-3922 | 0.014 | 493 | 503 | Neutral lipids |
| CellROX | Molecular Probes | C-10422 | 5 | 644 | 655 | ROS |
| Fluo-4 AM | Molecular Probes | F-14217 | 0.25 | 494 | 516 | Calcium |
| Hoechst 33342 | Sigma | B-2261 | 2.7 | 361 | 486 | Cell count |
| MitoSOX Red | Molecular Probes | M-36008 | 4 | 510 | 580 | Mitochondrial superoxide |
| PI | Sigma | P-4170 | 2.2 | 536 | 617 | Viability |
| TMRM | Molecular Probes | T-668 | 0.15 | 549 | 576 | MMP |
| YO-PRO-1 | Molecular Probes | Y-3603 | 1 | 491 | 509 | Apoptosis |

MMP: Mitochondrial membrane potential; PI: propidium iodide; TMRM: Tetramethyl rhodamine methyl ester.

**Supplementary Table S3. MEC for each parameter and AdCYP condition in HepG2 cells expressing a single CYP**

| **Drug** | **AdCYP** | **Apoptosis** | **MMP** | **ROS** | **Calcium** | **Mitochondrial superoxide** | **Lipids** | **TR** |
| --- | --- | --- | --- | --- | --- | --- | --- | --- |
| **Flutamide** | - | **500** | **500** | **500** | **500** | **500** | - | 1.2 |
|  | 3A4 1x | 500 | 750 | **250** | **250** | 500 | - | 2.5 |
|  | 3A4 4x | 250 | 500 | **125** | 250 | 250 | - | 5.0 |
|  | 3A4 10x | **125** | 250 | **125** | 250 | **125** | - | 5.0 |
|  | 2C19 1x | **250** | **250** | **250** | **250** | **250** | - | 2.5 |
|  | 2C19 4x | 250 | 250 | 250 | **100** | 250 | - | 6.2 |
|  | 2C19 10x | 100 | 250 | **50** | 75 | **50** | - | 12.4 |
|  | 1A2 1x | 250 | 500 | 500 | 500 | **75** | - | 2.5 |
|  | 1A2 4x | 250 | 500 | 500 | 500 | **50** | - | 12.4 |
|  | 1A2 10x | 125 | 500 | 250 | **50** | **50** | - | 12.4 |
| **Perhexiline** | - | 15 | 22.5 | 25 | **10** | **10** | **10** | 22 |
|  | 2D6 1x | 22.5 | 22.5 | >27.5 | **12.5** | 22.5 | >27.5 | 17.6 |
|  | 2D6 4x | 25 | 27.5 | >27.5 | **12.5** | 22.5 | >27.5 | 17.6 |
|  | 2D6 10x | 25 | >27.5 | >27.5 | **12.5** | 22.5 | >27.5 | 17.6 |
| **Tienilic acid** | - | 1200 | >1200 | 1200 | **800** | **800** | - | 10.3 |
|  | 2C9 1x | **600** | >1200 | 800 | **600** | **600** | - | 13.8 |
|  | 2C9 4x | **400** | >1200 | 600 | **400** | **400** | - | 20.7 |
|  | 2C9 10x | **200** | >1200 | 400 | **200** | 400 | - | 41.3 |
| **Troglitazone** | - | **100** | 200 | **100** | **100** | 200 | - | 6.4 |
|  | 3A4 1x | 100 | 200 | **50** | 75 | 200 | - | 12.8 |
|  | 3A4 4x | 75 | 200 | **50** | **50** | 75 | - | 12.8 |
|  | 3A4 10x | **50** | 150 | **50** | **50** | **50** | - | 12.8 |

**AdCYP**: Cells non-transduced (-) or individually transduced with three AdCYP doses to reach 1x, 4x or 10x of activity levels in human hepatocytes.

The lowest MEC (denoted in bold) was considered to calculate the TR.

**Supplementary Table S4. MEC for each parameter in HepG2 cells transduced with different combinations of adenovirus and exposed to model hepatotoxic drugs.**

**A) Isoniazid**

| **Adenovirus** | **Apoptosis** | **MMP** | **ROS** | **Calcium** | **Mitochondrial superoxide** | **TR** |
| --- | --- | --- | --- | --- | --- | --- |
| - | 30 | >50 | >50 | 30 | 30 | 0.25 |
| CYP2E1- 1X | 30 | >50 | 50 | 30 | 30 | 0.25 |
| CYP2E1-4X | 20 | >50 | 50 | 20 | 20 | 0.38 |
| CYP2E1-10X | 5 | >50 | 35 | 10 | 5 | 1.53 |
| CYP2E1-10X + GSTM1 | 30 | >50 | >50 | 20 | 30 | 0.38 |
| GSTM1 | 30 | >50 | >50 | >50 | >50 | 0.25 |

Cells non-transduced (-) or individually transduced with three Adv doses to reach 1x, 4x or 10x of activity levels in human hepatocytes.

.

**B) Acetaminophen**

| **Adenovirus** | **Apoptosis** | **MMP** | **ROS** | **Calcium** | **Mitochondrial superoxide** | **TR** |
| --- | --- | --- | --- | --- | --- | --- |
| - | 4 | 15 | 10 | 6 | 4 | 3.5 |
| CYP2E1-1X | 2 | 15 | 8 | 6 | 2 | 6.9 |
| CYP2E1-4X | 0.5 | 8 | 6 | 4 | 0.5 | 27.8 |
| CYP2E1-10X | 0.5 | 8 | 6 | 2 | 0.5 | 27.8 |
| CYP2E1-1X + CYP1A2-1X | 1 | 10 | 6 | 6 | 1 | 13.9 |
| CYP2E1-1X + CYP1A2-1X + GSTM1 | 4 | 15 | 15 | 15 | 6 | 3.5 |
| CYP2E1-4X + CYP1A2-4X | 0.5 | 6 | 0.5 | 0.5 | 0.5 | 27.8 |
| CYP2E1-4X + CYP1A2-4X + GSTM1 | 1 | 10 | 6 | 6 | 4 | 13.9 |
| GSTM1 | 4 | 15 | 15 | 10 | 6 | 3.5 |

**C) Valproate**

| **Adenovirus** | **Apoptosis** | **MMP** | **ROS** | **Calcium** | **Mitochondrial superoxide** | **Lipids** | **TR** |
| --- | --- | --- | --- | --- | --- | --- | --- |
| - | >12 | 10 | 12 | 6 | 6 | 6 | 8.0 |
| CYP2B6 -1X | >12 | 10 | 12 | 6 | 4 | 2 | 24.0 |
| CYP2B6-1X + CYP2C9-1X | 8 | 10 | >12 | 6 | 2 | 4 | 24.0 |
| CYP2B6 -–4X | 8 | 6 | 10 | 6 | 2 | 1 | 48.1 |
| CYP2B6-4X + CYP2C9-1X | >12 | 8 | >12 | 6 | 4 | 4 | 12.0 |
| CYP2B6-10X | 10 | 4 | 8 | 4 | 8 | 0.5 | 96.2 |
| CYP2B6-10X + CYP2C9-1X | 10 | 8 | >12 | 6 | 6 | 2 | 24.0 |

**D) Diclofenac**

| **Adenovirus** | **Apoptosis** | **MMP** | **ROS** | **Calcium** | **Mitochondrial superoxide** | **TR** |
| --- | --- | --- | --- | --- | --- | --- |
| - | 125 | 250 | 50 | 50 | 125 | 8.4 |
| CYP3A4-1X | 125 | 125 | 10 | 10 | 50 | 42 |
| CYP3A4-1X + CYP2C9-1X | 125 | 125 | 5 | 10 | 50 | 84 |
| CYP3A4-1X + UGT2B7 | 5 | 50 | 5 | 10 | 5 | 84 |
| CYP3A4-4X | 10 | 125 | 5 | 10 | 5 | 84 |
| CYP3A4-4X + CYP2C9-1X | 10 | 125 | 5 | 10 | 5 | 84 |
| CYP3A4-4X + UGT2B7 | 5 | 50 | 5 | 5 | 5 | 84 |
| CYP3A4-4X + CYP2C19-1X | 50 | 125 | 10 | 50 | 10 | 42 |

**Supplementary Figures**

**Supplementary figure S1. Cytotoxicity (A) and activity levels (B) of drug-metabolizing in HepG2 cells transduced with adenovirus.**

**Supplementary figure S2. Drug cytotoxicity in HepG2 cells individually transduced with CYP adenoviruses.**

**Supplementary figure S3. CYP activity levels in HepG2 cells co-transduced with CYP2C9 and CYP3A4 adenoviruses.**

**Supplementary figure S4. Heatmap of the toxic effects of amoxicillin/clavulanic acid in AdCYP-HepG2 cells.**


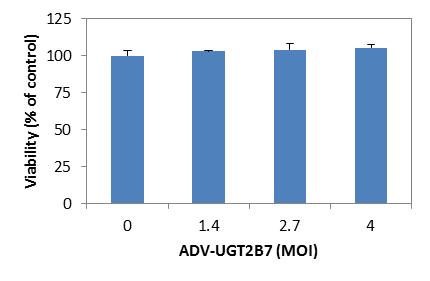

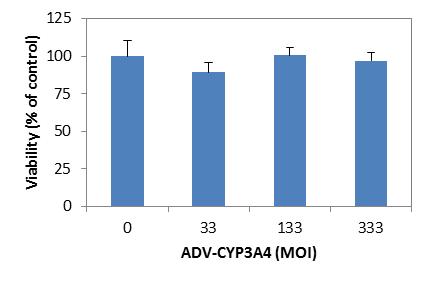

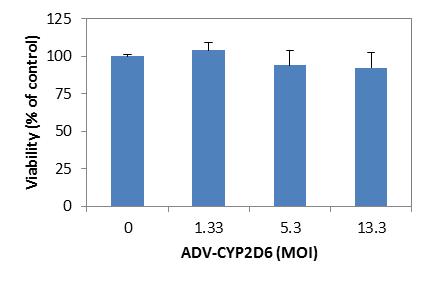

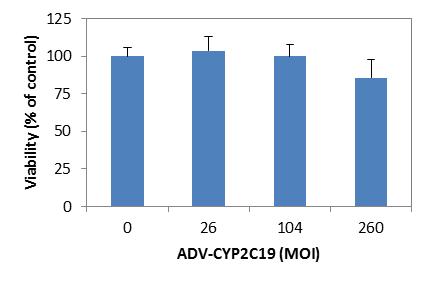

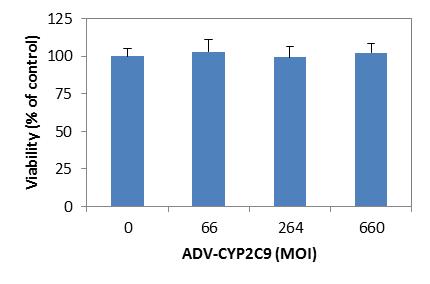

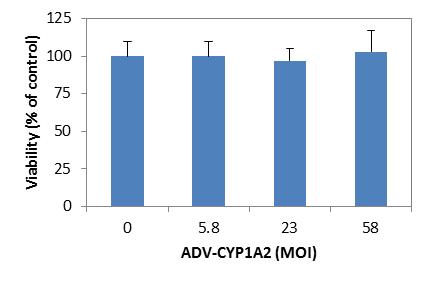

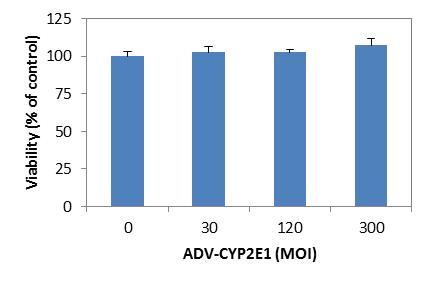

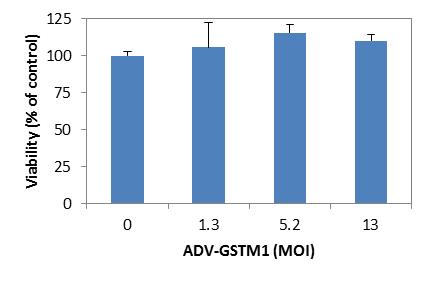

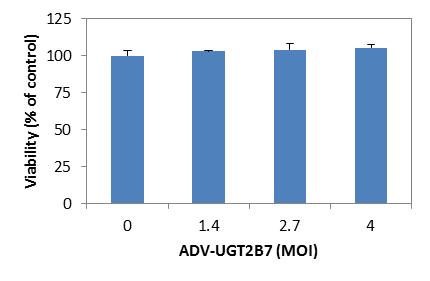


(A)


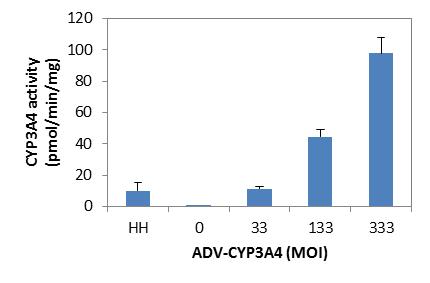

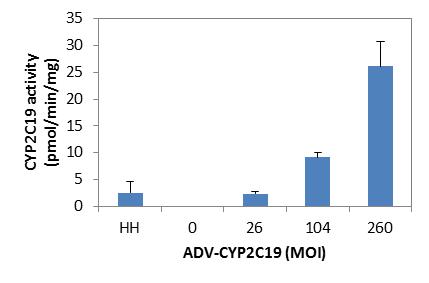

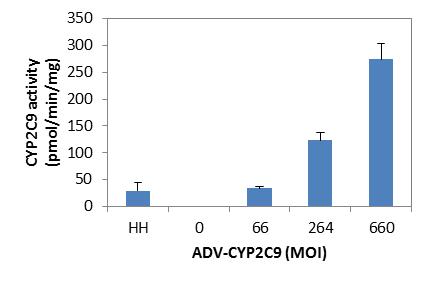

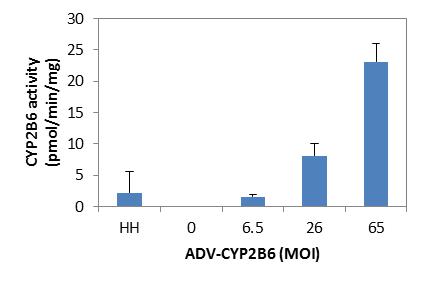

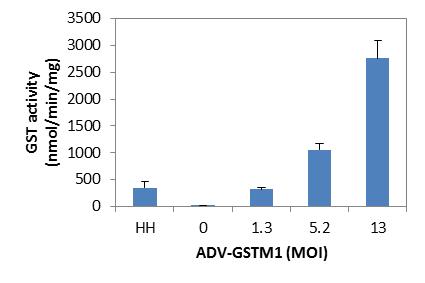

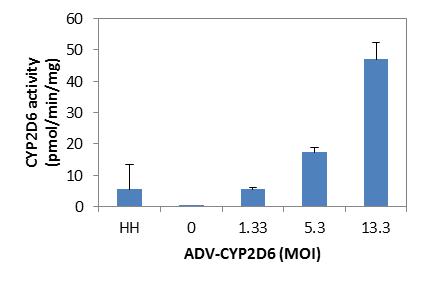

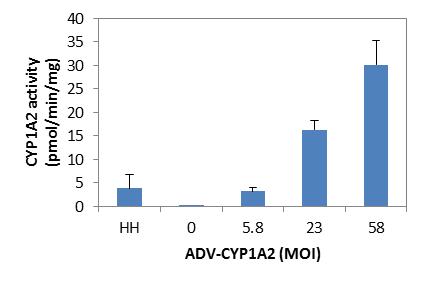

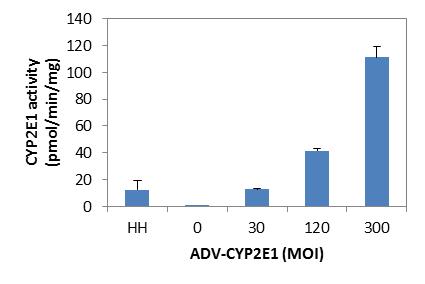

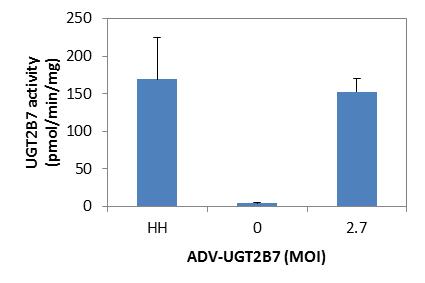


(B)

**Supplementary figure S1. Cytotoxicity and activity levels of drug-metabolizing in HepG2 cells transduced with adenovirus.** Cells were individually treated for 24 h with different doses (MOI) of a recombinant adenovirus for CYP3A4, CYP2C9, CYP2C19, CYP2B6, GSTM1 or UGT2B7. (A) The effects of each adenovirus construct on cell viability were evaluated 48 h later using MTT test and expressed of percentage of non-transduced HepG2 cells. (B) The corresponding enzyme activities were evaluated 48 h post-tranduction and compared to those of in control HepG2 cells and human hepatocytes (HH). Activity values for HH are from Tolosa et al 2016, Toxicol Sci 152: 214. Activity values of CYP3A4 (midazolam 1’-hydroxylation), CYP2C9 (diclofenac 4’-hydroxylation), CYP2C19 (S-mephenytoin 4’-hydroxylation), CYP2B6 (bupropion hydroxylation) and UGT2B7 (naloxone glucuronidation) were expressed as pmol of the product formed per minute and per mg of total cell protein and GST activity (1-Chloro-2,4-dinitrobenzene conjugation) was expressed as nmol of the product formed per minute and per mg of S9 fraction protein.

**Supplementary figure S2. Drug cytotoxicity in HepG2 cells individually transduced with CYP adenoviruses.** Cells transduced with distinct concentrations of a single AdCYP to reach 1x, 4x or 10x of activity levels of the corresponding CYP in human hepatocytes were treated for 24 h with increasing concentrations of model drugs. Then, cytotoxicity, assessed as loss of cell viability, was determined in AdCYP and in control HepG2 (C, non-transduced with adenoviruses) cells. Results are expressed as a percentage of the solvent-treated cells. The cytotoxicity of diclofenac, flutamide, isoniazid, paracetamol, perhexiline, tienilic acid, troglitazone and valproate in control and adCYP cells is exemplified. At least **p* < 0.05; ***p* < 0.01; ****p* < 0.005 as compared to control cells (Student's t test).

**

***

*

*

*

**

**

*

*

*

*

*

*

*

*

***

*

*

**

**

***

**

**

**

*

**

*

*

*

*

**


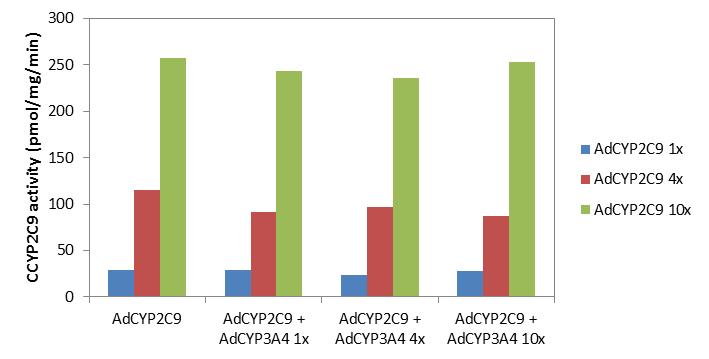

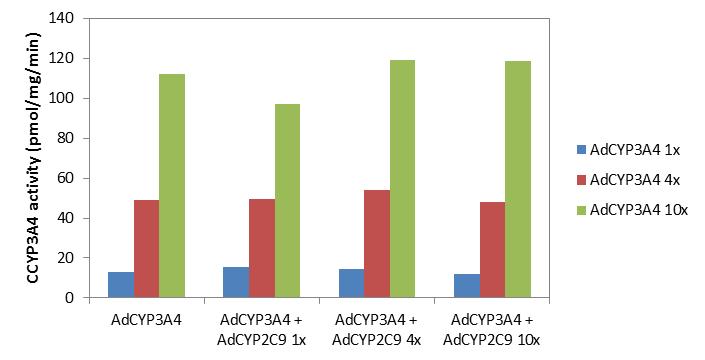


**Supplementary figure S3. CYP activity levels in HepG2 cells co-transduced with CYP2C9 and CYP3A4 adenoviruses.** Cells were transfected for 24 h with different mixtures of Ad-CYP2C9 and/or Ad-CYP3A4 and activities were evaluated 48 h later. (A) CYP2C9 activity (diclofenac 4’-hydroxylation) in cells transfected with different amounts of Ad-CYP2C9 to reach the same CYP2C9 activity (1x), 4-fold (4x) or 10-fold (10x) that human hepatocytes alone or in combination with increasing doses of Ad-CYP3A4 (equivalent to 1x, 4x or 10x of CYP3A4 activity in human hepatocytes). (B) CYP3A4 activity (midazolam 1’-hydroxylation) in cells transfected with different amounts of Ad-CYP3A4 to reach a CYP3A4 activity equivalent to 1x, 4x or 10x of that in human hepatocytes alone or in combination with different doses of Ad-CYP2C9 (equivalent to 1x, 4x or 10x of CYP2C9 activity in human hepatocytes).


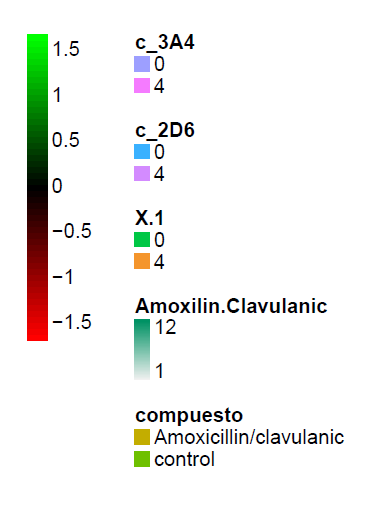

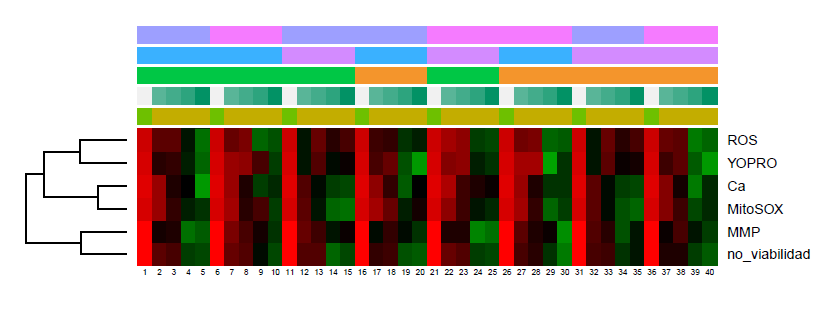


0

**Supplementary figure S4. Heatmap of the toxic effects of amoxicillin/clavulanic acid in AdCYP-HepG2 cells.**
